# Supplementary material for: Freewater estimatoR using iNtErpolated iniTialization (FERNET): Characterizing peritumoral edema using clinically feasible diffusion MRI data
Source: PLoS One. 2020 May 29;15(5):e0233645. doi: 10.1371/journal.pone.0233645 (PMC7259683; doi:10.1371/journal.pone.0233645)
Supplement: Attachment — Submitted filename: Fernet_Plos_reviews_final.docx [file pone.0233645.s001.docx]

**Response to Reviewers**

We would like to thank the reviewers for the comments and suggestions, which has helped improve the paper considerably. The paper has been revised, with extensive rewrite (as can be seen in the “tracked” document), and new experiments. Provided below are the response to the comments, with the reviewers’ comments being in black font and the responses being in red.

3. Have the authors made all data underlying the findings in their manuscript fully available?

The [PLOS Data policy](http://www.plosone.org/static/policies.action#sharing) requires authors to make all data underlying the findings described in their manuscript fully available without restriction, with rare exception (please refer to the Data Availability Statement in the manuscript PDF file). The data should be provided as part of the manuscript or its supporting information, or deposited to a public repository. For example, in addition to summary statistics, the data points behind means, medians and variance measures should be available. If there are restrictions on publicly sharing data—e.g. participant privacy or use of data from a third party—those must be specified.

Reviewer #1: No

Reviewer #2: Yes

We have used a freely available dataset, Philadelphia Neurodevelopmental Cohort (PNC), for some of our experiments on healthy controls. We cannot redistribute the dataset, but the link to the data has been provided in the paper in the Acknowledgements. The simulated data can be found at <https://github.com/DiCIPHR-Lab/Fernet/tree/paper_data>. The tumor data needs an IRB and can be provided to researchers on a collaborative basis. This has also been specified in the paper.

**Reviewer #1**

The work introduced a method to estimate free water volume fraction in the peritumoral region from single-shell diffusion MRI. The method aims to improve the results of a non-linear problem (two-compartment model) by using proper initialization. The manuscript is well-written and intelligible. The analyses and validations are robust and sufficient to support the authors' argument. This work could have an interesting contribution to a busy clinical setting.

We thank the reviewer for acknowledging the robustness of the work and the associated crucial clinical implications. We have provided detailed responses to the reviewer’s comments below.

There are some minor concerns that the authors should address in full:

1. The lambda_max in Eq.[4] is set to 2.5. As I understand, this value has to be smaller than d=3.0 so that the denominator is defined. Is there any reason behind the choice of 2.5, if not another value <3.0, say 2.9?

This is the upper bound of the range of axial diffusivity, and is the same number as chosen in 2009 by Pasternak. The range should encompass healthy WM but account for the possibility that pathology has increased axial diffusivity somewhat. This is now mentioned in the methods section where the choice of the upper bound was made (see line #s 113-115). The number is a heuristic and various numbers close to 2.5 were experimented with, with no effect. The number was retained as it was something with a precedent.

The above figure shows that values of lmax do not alter the initialization. On the top row, the MD map and initial tissue fraction (which is 1-VF) are shown. At top right, the difference in f_max values are shown in the blue voxels in periphery of ventricles, CSF, and necrotic tumor. Most voxels have zero difference and are not shown in blue. Voxels where f_max differs between the three values have initial tissue fraction values ~ 0.01, still much smaller than the value of f_max (~0.2) when lmax is 2.5. f_max is even larger when lmax is 2.9. The lmax parameter does not alter the initialization for values between 2.5 and 2.9.

2. During preprocessing, FA and MD maps were registered to the Eve atlas using non-linear registration. Since the atlas was built from healthy adults while the data in the experiment contain tumor, what strategy was used to ensure proper alignment and prevent registration error?

Registration is not needed to run our method on dataset. In our paper, healthy controls from Datasets 1 and 2 were registered to a common space. DTI scans from tumor patients were never registered to any other space. Rather, GLISTR (which is used for tumor and edema segmentation) was run on individual tumor cases to derive a mask of the edema region. Registration was undertaken only for the experiments on healthy data. We have corrected the methods at line #s 175-177 to reflect that only the healthy controls of Datasets 1 and 2 underwent registration. The previous draft had erroneously said that Dataset 3 was registered, which is a tumor dataset. This has now been corrected. Note that the order of the datasets has been changed, so that Dataset 2 is now the healthy controls and Dataset 3 are tumor cases. We have added a line to the Discussion (line #s 455-457) clarifying that registration is not required for the method.

3. In Fig.1, although the violin plots are informative and useful in showing the distribution of the error, it can only show one SNR value at a time (in panel A, B, and C). SNR=40 is optimistic and might not be found in some clinical data. Given the authors had results for other noise levels as well, could the heatmap of error be used with y-axis for SNR and x-axis for FW volume fraction

Figure 1 has been replaced with a new figure showing simulated data created by following a suggestion from Reviewer 2. We agree that an SNR of 40 is optimistic and have now elected to show our results at an SNR of 20. It is not possible to show multiple SNR values at a time, because we are trying to show both the center and the spread of the error in plotting for two methods. The analysis at various levels of SNR is now presented in Fig 1 D.

4. In Fig.1 A and B, the error (of FERNET) seems to increase with increased FW volume fraction, while in C, the errors seem to be stable. Is there any explanation for this observation?

This figure has now been replaced with the new Figure 1. We expect that the issue raised by the reviewer has been resolved.

5. Could the authors give some insights into why the 'bo-initialization' fails? In Fig.1 A, B, and C, besides higher error, in some cases, the distribution of the 'bo-initialization' results are scattered or skewed with outliers, which is not observed in FERNET results. Also, the trend of 'b0-initialization' results is harder to predict (overestimate in some cases but underestimate in other cases). A discussion on this would yield more insights into the problem.

This phenomenon is also reflected in Figures 2D, 5A, 5D. We believe that the combination of methodological choices in 2009 leads to this effect of “divergence” in the free water fit, which the authors had to resolve using spatial regularization. As our work shows, our choices in initialization have reduced the need for spatial regularization, and this effect can be seen in its behavior on synthetic data. This has now been added to the discussion (see line #s 430-439, 446-457).

6. The multi-shell ground truth used was from [18], which is a two-compartment model. I think NODDI is a better model to used as the ground truth given it also utilizes multi-shell data and has three compartments.

NODDI is a multi-shell model that has three compartments: a free water compartment, a zeppelin and orientation-dispersed sticks with a fixed axial diffusivity. The compartments of NODDI are thus not directly comparable to our method. The designed acquisition for NODDI involved very specific gradient strengths and values for big and small delta, which were optimized following (Alexander 2008) because the model relied on a priori fixed values for intracellular volume fraction, intrinsic free diffusivity and perpendicular extracellular diffusivity (Zhang 2012). Our data did not match this NODDI-optimized acquisition. Hoy’s method is the only method known to us that is directly comparable as it has the exact same two compartments. Hence, we believe that Hoy is a better model to compare to. This has now been added to the discussion (see line #s 423-429).

7. From Fig.2, seems like the FERNET always overestimates the FW volume fraction (which is consistent with Fig.1). Is there any explanation for this and potential method for correction?

The single-shell problem is ill-posed and lacks a closed form solution. As such, there is always a balancing act in the choices of parameters, as a result of which one compartment will always be either over or underestimated. It should also be noted that Hoy’s method itself may be biased, as there is no known ground truth. The aim of the paper was to obtain an initialization and free water estimation that could be best applied with tumor pathology. This provides a way to investigate large retrospective datasets for which only single shell information is available. This is expected to provide an insight into the effectiveness of and need for a free water model in pathology, motivating the way for a better acquisition in the clinic.

8. Diffusion MRI signal suffers from the degeneracy problem that isotropic diffusion (sphere) could have a similar signal compare to anisotropic diffusion with high dispersion (e.g. random oriented sharp tensor). I expect the peritumor region would have anisotropic diffusion with high dispersion since tumors could distort fibers pathway. This would be problematic as the solver could not distinguish between true free water diffusion and high dispersion anisotropic diffusion. Is this an issue in FERNET?

Yes, it is an issue with FERNET and any tensor model. NODDI and similar models which rely on multiple shells can estimate orientation dispersion, but this is not possible with single-shell data. Further resolution between isotropic diffusion and anisotropic diffusion with high dispersion has been shown to require new acquisition schemes such as spherical or double tensor encoding. Our method is intended to be able to work with a wide variety of clinically acquired data, and for getting a first characterization of peritumoral tissue from large retrospective databases, where the acquisition cannot be changed. As explained in the response above, any insights provided by our method on clinical data will motivate the way for better acquisitions, which can then distinguish between the scenarios that the reviewer has pointed out. Also, in the absence of peritumoral microenvironmental ground truth, this simpler model is an excellent preliminary characterization, before time is spent on lengthy acquisitions on very sick patients. We have added this to the Limitations section at line #s 495-497.

9. Single-shell clinical data usually has b=800, 1000, or 1500. Is the value of b-shell affect the estimation? For instance, free diffusion signal at b=1000 or 1500 is close to zero and more likely to be affected by the Rician noise floor than signal at b=800.

We show the comparison between b = 800 and 1000 in the figure below, on 15 controls with a b=800 acquisition (extracted from multi-shell data) and a b=1000 single-shell acquisition. There is little difference in the results. We do not have b=1500 data in order to test higher b values, but it is not a very traditional b-value that is used in the clinic.

**Reviewer #2:**

The authors proposed a method to improve free water estimation (FWE) in peritumoral regions with single-shell diffusion MRI data, by better initialization. The new initialization method took advantage of the image intensity in the b0 image to find the ratio between free water and tissue water. The paper presented a relatively comprehensive validation of the FERNET method with simulation data and experimental data in both healthy brains and tumor patients. The results look convincing and the topic potentially has an important impact for tumor research. I have several comments below.

We thank the reviewer for acknowledging the comprehensiveness of our presentation. We provide detailed responses to the comments and have extensively revised the paper to include the same.

1. Methods 2.2, Equation [2], I am not this equation tells an interpolation between two initialization strategies. Is that the “x” between f_b0 and f_MD stands for interpolation? Consider change the mathematical formulation.

The symbol x stands for multiplication and has been replaced with a “∙” which is a more suitable symbol. The formula is a logarithmic interpolation of two fractions, or it can be considered a linear interpolation of the log-transformations of the two fractions.

2. Since the FERNET heavily depends on the image intensity of the b0 image, errors can happen when b0 is problematic. 1) As the author mentioned, field inhomogeneity can affect the results and they used N4 correction. But it is know that N4 is not always successful. 2) Signal dropout due to susceptibility artifacts, e.g., in the frontal-orbital area. Please address these issues.

We appreciate the importance of bias correction, and hence propose it as a crucial step. However, bias correction has no known ground truth. N4 is the state of the art. Perfecting bias correction is outside the scope of this paper. Susceptibility will affect our method. Thus, we have added these to the Limitations at line #s 502-504.

3. f_b0 replies on the ratios between S0, Sw (CSF), and St(white matter). How about gray matter, how was the fitting done in the GM voxels?

DTI is primarily a WM modality and our model is specific to WM. Every voxel in the brain is fit using the signal estimated from white matter (St in Equation 3). We do not advocate relying on this model in GM. Results of the GM can be seen in Figures 2 and 4. In Figure 2, GM voxels fit in this way show a reasonable alignment with the multi-shell method of Hoy et al. However, in Figure 5, it can be seen that the most problematic areas of fit due to field inhomogeneity are in GM.

4. Some of the methodological details are quite empirical. For example, “we define as the 5th percentile of the unweighted signal in a region of WM, and we define as the 95th percentile of unweighted signal in a region of CSF”. How the percentiles were determined? Also, “the value of in Equation 2 is set following Equation 3”, I do not see rationale of determining this way. Equation 3 in itself is also empirical.

Fitting a multicompartment model to a single shell data is an ill-posed problem and cannot be solved without the imposition of heuristics. Each paper has their own (eg Pasternak 2009). Our thinking was that within any collection of WM voxels there will be partial voluming with CSF and GM. The 5^th^ percentile should minimize this effect. Similar reasoning was used in the choice of 95^th^ percentile for CSF. The choice of exponent in the interpolation step could also be considered another heuristic. The effect of this choice is described in the methods, lines 137-144. This was added to the Limitations.

5. Was regularization performed in this study and how? The authors mentioned in Results 3.4, that the proposed method was less dependent on regularization. This needs to be clarified.

It was an in-house regularization in MATLAB from one of the authors (Pasternak) which was used in his 2009 paper. Regularization helped the Pasternak method, but did not improve the new initialization that we proposed, as is shown in Figure 3.

6. Method 2.3 “0_ and 0_ are values taken as reference from selected voxels in human data”. Where were the selected voxels? I think it is much better to do a segmentation of the b0 image to obtain S0 in WM, GM, and CSF.

In agreement with the reviewer’s suggestion, we have changed the simulation portion of the methods extensively. Figure 1 is now replaced with a new figure. The method of deriving the “ground truth” values of B0, tensor eigenvalues, MD, and FA are described in line #s 201-219, and the segmentation masks are displayed in Figure 1 next to each simulation. Since our method is intended to be used in WM and the peritumoral region of tumor patients, we did not simulate GM voxels, as suggested.

7. Method 2.4.1 “Automatic tumor and peritumoral region segmentation was obtained in the patients from Dataset 1 and Dataset 2 using GLISTR”. Please access the segmentation accuracy, at least in part of the data.

Segmentation of tumor and edema are not the crux of the paper. These could be manually drawn, or many tools available (like CAPTk, DeepMedic) can be used. We used a very well-established tool, whose accuracy has been validated by comparing to those segmentations produced by clinical experts and two state-of-the-art methods (Ref. 25 in the paper). Testing the accuracy of segmentation is beyond the scope of the paper, and not relevant to the results.

8. Results 3.2, in the simulation, both b0 initialization and interpolated initialization results showed that the estimation errors increased as the ground truth free water fraction increased. Please explain.

Due to changes in the simulation method, the results now show that errors with interpolated initialization decrease as the ground truth VF is increased. However, we do not know the ground truth VF of the healthy data which we derived the ground truth values of B0, FA, and MD from. It is likely to be in between the values of 0 (Fig 1A) and 0.15 (Fig 1B), but this is not known. We have presented scenarios that emulate the combinations that we see in real life (healthy, edematous).

9. Figure 2, it is counterintuitive that high correlation was shown in green, low correlation was shown in red, and negative correlation is shown in blue.

We agree. The correlation maps Figure 2 have now been changed.

10. In Figure 5, should (A) and (B) be switched? It looks like that the percentage of unrealistic fit is higher with bias field correction. Also, some WM regions in this figure were white, which is out of the colorbar. What does that indicate?

The reviewer is correct that the images in the figure were switched. The figure (now Figure 4) has been corrected. In addition, the caption has been updated to reflect the fact that those voxels where the value was zero are not plotted, so that the underlying structure of the template image can be seen.

11. To test the accuracy of fiber tracking, it would be better to do the validation with the multi-shell data and compare the results with multi-shell FWE.

Our tractography figure is simply meant as an illustration of the power of our method over current state of the art tensor-based methods on single-shell data commonly used in the clinic, that is, how it improves the tractography in the edema, even in the case of the simplest tracking methodology, deterministic, that is used in the clinic. Exploring multiple tractography algorithms are beyond the scope of this paper and will be undertaken in future work. That will also involve comparing the tracking improvement from multi-shell free water estimation. However, at the outset it may be noted, that having an advanced multi-shell acquisition will also facilitate the fitting of a higher order model instead of tensors, which will lead to additional improvement in tractography. However, exploring all these nuances is beyond the scope of the current paper. We have added to the Discussion in the paper and suggested this as future work (line #s 514-518).
